# Mental health challenges faced by young medical residents fighting COVID-19 in tunisia

**DOI:** 10.1192/j.eurpsy.2021.766

**Published:** 2021-08-13

**Authors:** M. Dhemaid, W. Abbes, F. Ellough, A. Bezzaouia, S. Hafi, L. Ghanmi

**Affiliations:** 1 Psychiatry, regional hospital of Gabes, Gabes, Tunisia; 2 Prehospital Emergency Care Service 05, regional hospital of Gabes, Gabes, Tunisia

**Keywords:** young medical residents, Depression, Anxiety, COVID-19 outbreak

## Abstract

**Introduction:**

COVID-19 pandemic affected not only physical health of individuals, and communities but also their mental health worldwide. Young physicians, who were providing care for patients during the outbreak in a global atmosphere of stress, anxiety and depression, were not spared.

**Objectives:**

To assess anxiety and depression among young medical resident exposed to COVID-19 in Tunisia and its associated factors

**Methods:**

It was a cross-sectional, descriptive and analytical online-based survey, from April 19, 2020, to May 5, 2020 on 180 medical residents in training, via a Google-Form link. We used a self-administered anonymous questionnaire containing sociodemographic and clinical data. Hospital Anxiety and Depression Scale (HAD) validated in the Tunisian dialectal version was used to assess anxiety and depression.

**Results:**

Among 180 young doctors included, 70.2% were female, 16% were married, 81.8% worked in a university Hospital and 51.1% were frontline caregivers involved in primary screening. Our study revealed that 66.1% of young medical residents were suffering from anxiety (severe anxiety: 28.9 %) and 61.7% of them from depression (severe depression: 29.1%). Anxiety disorder was correlated to female gender (p=0.008), being married (0.001), worse quality of sleep (p<10^-3^) and increased consumption of tea and coffee (p=0.012). Depression was associated to worse quality of sleep (p<10^-3^), lack of physical activity (p<10^-3^), shortage of personal protective equipment (p=0,027) and anxiety disorder (p<10^-3^).

**Conclusions:**

Our study indicated a high proportion of anxiety and depression among young doctors in training, needing systematic screening in order to prevent them.

